# Unanticipated population structure of European grayling in its northern distribution: implications for conservation prioritization

**DOI:** 10.1186/1742-9994-6-6

**Published:** 2009-03-30

**Authors:** Akarapong Swatdipong, Anti Vasemägi, Mikko T Koskinen, Jorma Piironen, Craig R Primmer

**Affiliations:** 1 Department of Biology, University of Turku, Turku, Finland; 2 Finnzymes Diagnostics, Espoo, Finland; 3 Finnish Game and Fisheries Research Institute, Joensuu, Finland

## Abstract

**Background:**

The European grayling (*Thymallus thymallus*) is a salmonid fish native to Europe, with a distribution ranging from England and France to the Ural Mountains of north-western Russia. The majority of grayling populations inhabit freshwater rivers and lakes but some populations also occupy brackish water in northern parts of the Baltic Sea. Previous population genetic studies have demonstrated that grayling populations in Finland, Estonia and Russia belong to a single mitochondrial lineage and exhibit high levels of differentiation even at a small geographic scale. As a result, we predicted that grayling populations should not cluster regionally. Despite the extensive amount of genetic research that has been carried out on grayling, comprehensive national-level information on population structure of grayling in Northern Europe is still lacking. Yet this is the level at which populations are currently managed.

**Results:**

We found unanticipated population structure of grayling clustering into three groups largely corresponding to the northern, Baltic and south-eastern geographic areas of Finland using 13 microsatellite loci. We also found a high level of genetic differentiation among the groups and moderate to high differentiation within the groups. This combined with low variability strongly indicates that genetic drift and limited migration have a major impact on grayling population structure. An allele size permutation test indicated that mutations at microsatellite loci have not significantly contributed to genetic differentiation among the three Finnish groups. However, at the European scale, mutations had significantly contributed to population differentiation.

**Conclusion:**

This research provides novel genetic information on European grayling in its northern distribution range and has clear implications for supporting country-scale conservation efforts. Specifically, the strong between population divergence observed indicates that single populations should generally be recognized as separate management units. We also introduced an alternative prioritization strategy for population conservation based on the evaluation of the relative roles of different evolutionary forces shaping the gene pools. We envision that the proposed approach to categorize populations for conservation will be a useful tool for wildlife researchers and conservationists working on a diverse range of organisms.

## Background

The European grayling, *Thymallus thymallus*, is a salmonid fish highly appreciated by recreational anglers. It is native to Europe with a distribution from England and France to the Ural Mountains of north-western Russia. It can inhabit stream, riverine or lacustrine habitats and show large phenotypic differences in gill raker number, body size, weight at first spawning and fecundity [1]. While grayling occur mostly in freshwater habitats, some populations also occupy brackish water in the northern parts of the Baltic Sea.

Population genetic structure of the European grayling has been studied rather extensively across its natural distribution, but particularly in central Europe, using both mitochondrial sequences and microsatellite markers [2-7]. In central and northern Europe, the population structure is represented by four major lineages with (lineage) contact zones in Germany [8] and northern Sweden and Finland [9]. In northern Europe, two major lineages have been described (3.1% and 1.1% divergences using mtDNA PCR-RFLP and 529-bp of ND5 sequence, respectively; [9]). One lineage that almost exclusively inhabits Sweden and Norway most likely originated from a central European refugium while grayling in Finland, Estonia and north-western Russia belong to a different mitochondrial lineage originating most likely from an eastern European refugium [9]. Northern European grayling have been studied also at a very small geographic scale [10]. In lake Saimaa, Finland, Koskinen *et al*. [11] found evidence of severely limited gene flow among populations separated by just tens of kilometres.

Despite the quite extensive amount of genetic research that has been carried out on grayling, detailed national-level information of grayling population structure in Northern Europe is lacking. Yet, this is the level at which populations are currently managed. Based on the previous work, we expect that grayling populations in Northern Europe exhibit high genetic differentiation even at a small geographic scale (*prediction 1*) and possibly show just a relatively weak isolation-by-distance signal as genetic drift should be the dominant evolutionary force compared to migration. However, as Finnish grayling populations belong to a single mitochondrial lineage on one hand and show extremely low gene flow on the other, we do not expect that grayling in Finland exhibit further genetic clustering or grouping (*prediction 2*).

In this study, the major objective was to provide genetic information from a geographically representative set of populations to assist country-scale conservation of grayling in Finland. We analyzed grayling sampled throughout Finland in order to test the predictions based on the knowledge from previous population genetic studies, using a panel of 13 microsatellite markers. For comparative purposes, we analyzed grayling populations from Russia, Sweden, Norway and Germany. We also categorized and prioritized populations according to predominant evolutionary forces, providing useful information for the development of a scientifically justified national-scale conservation strategy of grayling in Finland.

## Materials and methods

### Fish samples

**Figure 1:**
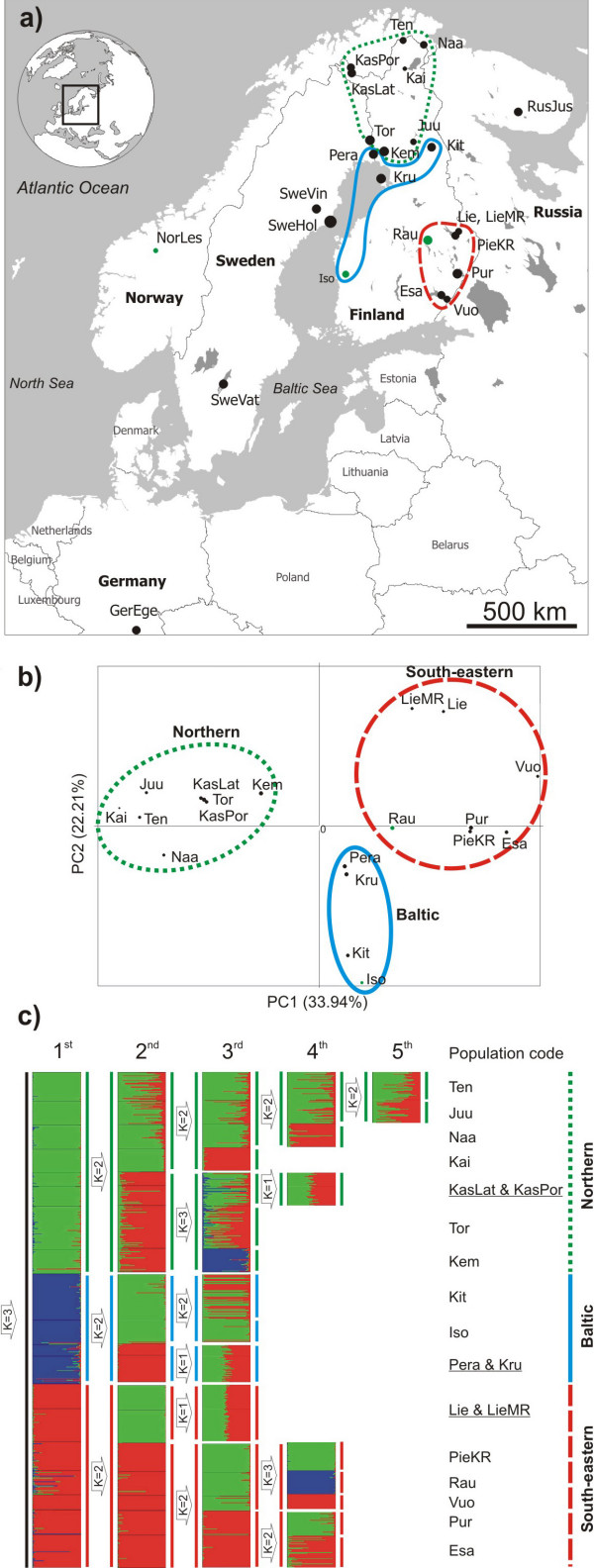
**Grayling sampling locations and analyses of population structure based on 13-microsatellite loci**. Populations are coded as in table 1. a) A map indicating sampling locations. Three population groups are indicated with different color lines corresponding to the three clusters identified using PCA. Dot sizes are proportioned to allelic richness of each population. Black and green dots stand for indigenous and supplementary stocked populations, respectively. b) Principal Component Analysis of Finnish grayling populations based on microsatellite allele frequencies. c) Individual clustering as inferred by series of hierarchical partitioning using Structure. Each individual is represented by a thin horizontal line pooled into K-colored blocks indicating individual's membership fractioned in K clusters. Black horizontal lines separate individuals from different sampling sites.

Grayling were sampled from 15 locations within Finland, between 1996 to 2001 (figure 1a). Fin clips were stored in 95% ethanol. DNA was extracted using a salt-based method described by Aljanabi and Martinez [12]. Information on population status, location and geographic coordinates are given in table 1.

**Table 1:** Locations and status of grayling populations in the study.

Location						
		
Water system	Code	Region	Co-ordinates		Source status	Sampling site
Tenojoki	Ten	Finland. N	69 54' 40.97"N	27 02' 48.36"E	indigenous	river
Näätämöjoki	Naa	Finland. N	69 42' 22.98"N	29 00' 05.76"E	indigenous	river
Kaitamo (Inari)	Kai	Finland. N	68 47' 12.94"N	26 58' 39.83"E	indigenous	lake
Käsivarsi, Poroeno	KasPor	Finland. N	68 58' 46.33"N	22 04' 36.37"E	indigenous	river
Käsivarsi, Lätäseno	KasLat	Finland. N	68 50' 00.00"N	22 11' 52.34"E	indigenous	river
Tornionjoki	Tor	Finland. N	66 30' 00.00"N	23 44' 15.16"E	indigenous	river
Juujarvi	Juu	Finland. N	66 22' 41.59"N	27 16' 50.43"E	indigenous	river
Kemijoki	Kem	Finland. N	66 06' 40.75"N	24 51' 58.33"E	indigenous	river
Kitkajärvi	Kit	Finland. N	66 10' 02.44"N	28 42' 32.28"E	indigenous	lake
Perämeri	Pera	Finland. N	66 01' 35.99"N	24 00' 47.04"E	indigenous	sea
Ulkokrunnit	Kru	Finland. Bothnian bay	65 12' 23.62"N	24 35' 14.31"E	indigenous	sea
Lieksanjoki	Lie	Finland. SE	63 18' 43.44"N	30 02' 16.60"E	indigenous	river
Lieksanjoki	LieMR	Finland. SE	63 18' 43.44"N	30 02' 16.60"E	indigenous	river
Pielinen	PieKR	Finland. SE	63 12' 45.27"N	29 46' 19.54"E	indigenous	lake
Rauanjoki	Rau	Finland. SE	63 07' 17.52"N	27 46' 44.04"E	hatchery	river
Isojoki	Iso	Finland. SW	62 06' 36.58"N	21 57' 21.28"E	mixed	river
Puruvesi	Pur	Finland. SE	61 57' 07.38"N	29 37' 19.22"E	indigenous	lake
Etelä-Saimaa	Esa	Finland. SE	61 19' 32.54"N	28 23' 22.52"E	indigenous	lake
Vuoksi river	Vuo	Finland. SE	61 10' 17.28"N	28 46' 44.43"E	indigenous	river^a^
Juzija river	RusJus	Russia. Kola peninsula	66 58' 13.80"N	36 20' 48.86"E	indigenous	river
Vindelälven	SweVin	Sweden. E	64 11' 58.34"N	19 42' 07.67"E	indigenous	river
Holmön	SweHol	Sweden. Bothnian bay	63 47' 59.63"N	20 51' 28.83"E	indigenous	sea
Vättern	SweVat	Sweden. S	58 17' 41.85"N	14 28' 39.98"E	indigenous	lake
Lesjaskogsvatn	NorLes	Norway. S	62 12' 02.67"N	8 25' 15.11"E	introduced	lake
Eger river	GerEge	Germany. Central	49 27' 11.74"N	11 04' 21.96"E	indigenous	river

^a ^The outlet of lake Saimaa.

### Microsatellite data

Initially, seventeen microsatellite markers employed by Koskinen *et al*. [13] were re-screened using 16 individuals representing a number of populations and two loci were then excluded (BFRO9 and BFRO16) due to low levels of polymorphism. Forward primers were labeled fluorescently with FAM for BFRO4, BFRO5, BFRO7, BFRO10 and BFRO13; VIC for BFRO15, BFRO17, BFRO18 and Ogo2; NED for BFRO12, Cocl23 and Str85INRA; and PET for BFRO11, ONE2, and Str73INRA. Primer concentrations were optimized for co-amplification of 15 loci in a single multiplex PCR and the amplicons were size-measured in a single capillary electrophoresis. The 6.25 μl multiplex PCR reaction consisted of ca. 125 ng of template DNA, 1× multiplex PCR master mix (Qiagen) and 0.015 to 1.915 μM of each primer. Exact concentrations of primers are provided in the supplementary material [see Additional file 1]. Amplifications were carried out in a PTC100 thermal cycler (MJ Research) with an initial heat-activation at 95°C for 15 minutes (min) followed by 37 cycles of denaturation at 94°C for 30 seconds (sec), annealing at 55°C for 90 sec and extension at 72°C for 60 sec. The PCR was terminated after 30 min of final extension at 60°C.

PCR products were diluted, denatured and then electrophoresed on an ABI Prism 3130xl genetic analyzer (Applied Biosystems/Hitachi) along with GeneScan 600 LIZ size standard (Applied Biosystems). DNA fragments were genotyped using GeneMapper 4.0 (Applied Biosystems). All genotypes were manually inspected and examples of electropherograms are shown in the supplementary material [see Additional file 2]. Besides the 15 newly genotyped Finnish populations, previously published data [13] from seven populations within Finland and six populations from Russia, Sweden, Norway and Germany were included (table 1). Three of the previously published Finnish populations (102 individuals in total) were re-genotyped in this study to enable calibration of allele sizes.

### Statistical analyses

#### Microsatellite diversity, Hardy-Weinberg and genotypic linkage equilibrium

GenePop 3.4 [14] was initially employed to screen for deviations from Hardy-Weinberg (H-W) equilibrium and two loci (BFRO4 and ONE2) were excluded due to highly significant heterozygote deficiency (P < 0.0001) in several populations, likely indicating the presence of null alleles. Thus, the final dataset comprised 13 loci. Because some of the sampled populations can be affected by stocking with individuals of non-native origin (table 1), Structure 2.2 [15] was used to identify individuals that represented genotypes highly unlikely to be native to the sampled population. In total, 15 individuals belonging to four populations (1–6 individuals per population) were identified with highly unlikely genotypes compared to the rest of the samples and these putatively non-natives were excluded from further analysis. The final dataset consisted of 936 individuals from 25 populations.

Allele number and allelic richness were estimated using FSTAT 2.9.3.2 [16]. Observed and expected heterozygosity were measured using Microsatellite Toolkit 3.1 [17]. Deviations from Hardy-Weinberg equilibrium and genotypic linkage equilibrium were calculated using Genepop 3.4. For the combination of separate tests across loci (H-W test) or locus pairs (linkage equilibrium test), Fisher's procedure was applied [18]. Throughout the study, sequential Bonferroni corrections [19] were performed to correct for multiple testing.

#### Genetic differentiation and relationships between populations

Inter-population genetic divergence was calculated using the multilocus *F*_ST _estimator of Weir & Cockerham [20] with FSTAT 2.9.3.2. Population differentiation was tested as genic differentiation for all population pairs using Genepop 3.4. The genetic relationship among populations was examined by Principal Component Analysis (PCA), based on allele frequencies, using PCAGEN 1.2 [21]. Population clustering was performed with a hierarchical partitioning approach as previously employed by Vähä *et al*. [22], using Structure 2.2 based on the delta K method [23] calculated from 20 replicates of ln Pr(X|K) under each K. Different run lengths were used (burn-in 20 000 to 80 000 iterations and, after that, data were collected for 20 000 to 80 000 iterations) to achieve stable results.

#### Analysis of molecular variance

To estimate the amount of molecular variation associated with different sets of population groupings, hierarchical analysis of molecular variance (AMOVA) was performed using Arlequin 3.1 [24]. By this analysis, genetic variation residing within each alternative population clustering was measured. Three alternative scenarios were tested based on the PCA results: grouping 1 (three population groups corresponding to northern, Baltic and south-eastern populations), grouping 2 (northern vs Baltic and south-eastern groups combined) and grouping 3 (Pera and Kru were re-located into the south-eastern group. Other populations were as in grouping 1).

#### Mantel test

An association between the geographic distance and genetic divergence matrices was examined using the Mantel test implemented in the GenAlEx 6.1 package [25] supported by multiple regression and a correlation extension procedure [26]. The statistical significance of the parameter estimates was obtained via 9 999 permutations. Inter-population geographic distances were directly calculated from latitude and longitude data using the great circle distance method [27]. *F*_ST_/(1-*F*_ST_) was used as a measurement of genetic divergence in the Mantel test while the geographic distance matrix was used as ln transformed as well as raw distance [28]. For detecting IBD within the three population groups with higher statistical power, a so-called "pooled within-stratum" approach was applied to the Mantel test as suggested by Smouse, PE (personal communication). Briefly, matrices from each of three population groups were permuted using GenAlex 6.1 to generate 999 values of the sum of permuted XY (spXY). The spXY from each population group were resampled using PopTool 2.7 [29] to yield 1 000 random combinations of "pooled" spXY [*sum*(*XY*) = *sumA*(*XY*) + *sumB*(*XY*) + *sumC*(*XY*); A, B and C stand for each population group]. Later, "pooled" sum(X^2^) and sum(Y^2^) were calculated accordingly. One-thousand pooled R_XY _were subsequently obtained as . The pooled R_XY _were ranked and the corresponding P-value was calculated.

#### Allele size permutation test

To test whether stepwise-like mutations have significantly contributed to genetic differentiation between populations, the allele size randomization test [30] was employed using SPAGeDi 1.2 [31]. Theoretically, *R*_ST _is analogous to *F*_ST_, where *R*_ST _is a stepwise mutation based measurement of genetic differentiation taking into account the variance of microsatellite allele size [32]. When the contribution of mutations to genetic differentiation is not significant as compared with genetic drift and migration *F*_ST _and *R*_ST _are expected to have similar values. If stepwise-like mutations have significantly contributed to the differentiation *R*_ST _is expected to be larger than *F*_ST_. In the allele size permutation test, the distribution of *R*_ST _values from 10 000 permutations (p*R*_ST_) was compared to observed *R*_ST_. The test of allele size permutation was performed at three different hierarchical levels; among population groups within Finland as identified by PCA and Structure analyses, among countries and among 25 grayling populations.

#### Population prioritization for conservation

In order to rank populations in terms of their conservation priority, Contrib 1.20 [33,34] was used to calculate both the diversity (allelic richness) and the differentiation (related to Nei's *D*_ST _and *G*_ST_) components of each population. These parameters are relative measurements and are evaluated as the contribution of each population to total allelic richness (contribution to total allelic richness; CTR) pooled from all populations. Thus, negative values indicate that the diversity or the differentiation of a population is lower than the mean of the whole dataset. For prioritizing grayling populations within Finland, foreign populations were excluded from the analysis in order to avoid the underestimation of the differentiation component within Finnish populations. Two Finnish populations (Iso and Rau) were also excluded because of their mixed origin, from hatchery stocking of non-native fish [35], which would artificially increase their diversity component compared to indigenous gene pools.

## Results

### Microsatellite diversity, Hardy-Weinberg and genotypic linkage equilibrium

The average number of alleles per locus varied from 1.6 (Kai) to 5.4 (Tor) while the average allelic richness ranged from 1.6 (Kai) to 4.6 (SweHol; table 2). Observed and expected heterozygosity were lowest in Kai (H_o _= 0.21; H_e _= 0.20) and highest in SweHol (H_o _= 0.60; H_e _= 0.63). No deviation from Hardy-Weinberg equilibrium was observed for any locus after the sequential Bonferroni correction (*k *= 13). Genotypic linkage equilibrium tests suggested linkage disequilibrium (P < 0.0001) between five population-locus pairs: BFRO7-BFRO13 in Esa; BFRO11-BFRO13 and BFRO13-BFRO18 in Kit; BFRO12-Cocl3 and BFRO15-Cocl23 in Juu. However, as each of these five linkage disequilibrium occurred only in a single population and none involved the same two loci, these loci are likely not in physical linkage. Beside these five locus pairs, no deviation from genotypic linkage equilibrium was observed after the sequential Bonferroni correction (*k *= 78; P = 0.0015–0.9999). Therefore, for subsequent analyses, all loci were assumed to have independent segregation of alleles.

**Table 2:** Locations, sample sizes, microsatellite diversity estimates and Hardy-Weinberg (H-W) equilibrium test for the grayling.

Location			Microsatellite diversity^a^			
			
Water system	Code	Sample size	A_r_	H_o_	H_e_	H-W^b^
Tenojoki	Ten^c^	42	2.68 (1–5)	0.27	0.31	0.1941
Näätämöjoki	Naa	35	2.79 (1–7)	0.33	0.36	0.2232
Kaitamo (Inarijoki)	Kai	34	1.56 (1–3)	0.21	0.20	0.9783
Käsivarsi, Poroeno	KasPor	20	3.10 (1–7)	0.31	0.33	0.0436
Käsivarsi, Lätäseno	KasLat^c^	29	3.22 (1–6)	0.36	0.35	0.9356
Tornionjoki	Tor^c^	63	3.64 (1–7)	0.36	0.37	0.2143
Juujarvi	Juu	35	2.47 (1–5)	0.29	0.27	0.6661
Kemijoki	Kem	35	3.63 (2–6)	0.47	0.47	0.5617
Kitkajärvi	Kit	67	3.20 (1–6)	0.46	0.49	0.0597
Perämeri	Pera	17	3.44 (1–6)	0.47	0.47	0.9974
Ulkokrunnit	Kru^c^	40	3.55 (1–6)	0.48	0.48	0.1999
Lieksanjoki	Lie	36	2.44 (1–5)	0.39	0.36	0.7229
Lieksanjoki	LieMR^c^	48	2.58 (1–4)	0.35	0.33	0.9241
Pielinen	PieKR^c^	42	3.02 (1–5)	0.47	0.47	0.0615
Rauanjoki	Rau	35	3.43 (1–6)	0.52	0.52	0.0039
Isojoki	Iso	36	2.61 (1–5)	0.41	0.44	0.1002
Puruvesi	Pur	36	3.55 (2–5)	0.52	0.53	0.0056
Etelä-Saimaa	Esa^c^	48	2.98 (1–5)	0.49	0.47	0.1214
Vuoksi river	Vuo	22	2.51 (1–4)	0.36	0.34	0.6244
Juzija river	RusJus^c^	32	3.12 (1–6)	0.41	0.42	0.8362
Vindelälven	SweVin^c^	38	3.37 (1–6)	0.46	0.47	0.4106
Holmön	SweHol^c^	34	4.55 (2–7)	0.60	0.63	0.3041
Vättern	SweVat^c^	45	3.32 (1–8)	0.43	0.45	0.0170
Lesjaskogsvatn	NorLes^c^	30	1.81 (1–4)	0.22	0.24	0.4477
Eger river	GerEge^c^	37	3.23 (1–6)	0.41	0.41	0.0353

^a ^allelic richness (based on 16 individual re-samplings; A_r_), observed heterozygosity (H_o_) and expected heterozygosity (H_e_) within a population across the 13 microsatellite loci. Numbers within parentheses indicate range for A_r_. ^b ^P-value for H-W equilibrium test across 13 loci, no populations remained significant following the sequential Bonferroni correction. ^c ^Data taken from Koskinen *et al*. [13].

### Level of differentiation and genetic relationships between populations

When all 25 populations were included in the PCA, three populations (GerEge, Norles and SweVat) were very distinct compared to the rest along the first principal component axis (data not shown). This deep divergence corresponds to two mtDNA lineages described by Koskinen *et al*. [9]. When only 19 Finnish populations were included in the analyses, populations clustered into three separate groups: 'northern', 'Baltic' and 'south-eastern' (figure 1b). The 'northern' group was the most homogeneous and distinct from the latter two groups. The 'northern' group included eight populations, three of them (Ten, Naa and Kai) inhabiting rivers flowing north to the Barents Sea basin, four of them (KasLat, KasPor, Tor and Kem) to the Baltic Sea basin and Juu to the White Sea basin. In the 'south-eastern' group, both populations from Lieksanjoki (Lie and LieMR) were clustered closely to each other but showed some distance from the remaining south-eastern members. The third, 'Baltic', group was separated from the two other population groups along the second PCA axis and consisted of four populations, including Kit that is close to Iso. This was surprising, as Kit is currently 174 km from the Baltic Sea and 556 km from Iso. Additionally, the two Swedish populations (SweHol and SweVin) from the Baltic coastline initially clustered with the 'Baltic' group and the Russian population from the Kola Peninsula (RusJus) clustered with the 'northern' group (data not shown).

Similar to the PCA results, population clustering analysis using the program Structure without prior information about population of origin of individuals initially distinguished three main groups of Finnish grayling corresponding to 'northern', 'Baltic' and 'south-eastern' groups (figure 1c). After the second round of partitioning, six separate clusters were identified. The third partitioning revealed eleven clusters. Kai, Tor, Kem, Kit and Iso were separated from the remaining samples, while the pairs of Pera-Kru and Lie-LieMR exhibited no further separation. After the fourth and fifth partitioning, the northern and south-eastern groups were divided into seven and six clusters, respectively. In total, the hierarchical partitioning series produced sixteen separate clusters corresponding very well to the number of geographic sampling sites (eighteen Finnish sampling sites in total).

The global *F*_ST _across all 25 populations was 0.342 indicating a very high level of differentiation among individual grayling populations. Within Finland, the global *F*_ST _was 0.294 and the *F*_ST _within the three population clusters was 0.145, 0.146 and 0.256 for the northern, Baltic and south-eastern group, respectively. Pairwise *F*_ST _among populations is detailed in the supplementary material [see Additional file 3]. The test of genic differentiation indicated that all pairs of populations were significantly different to each other (P < 0.001), except for KasPor-KasLat and Pera-Kru (P = 0.259 and 0.427, respectively).

The hierarchical analysis of molecular variance revealed that the proportion of total genetic variance due to differences between groups was highest for grouping 1 (*F*_CT _= 0.191). The two alternative hierarchical groupings explained smaller proportions of variation (*F*_CT _= 0.183 and 0.160 for grouping 2 and 3, respectively).

### Mantel test

**Figure 2:**
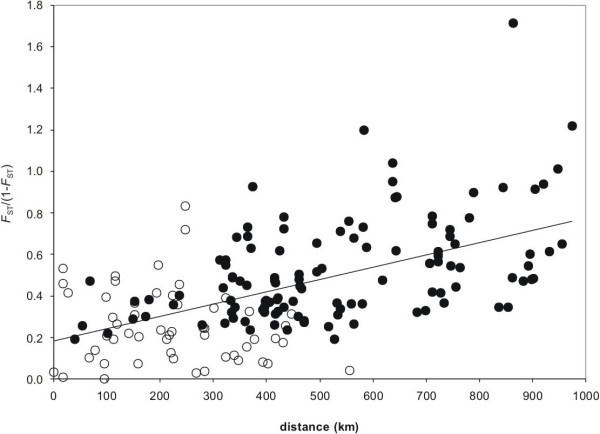
**Mantel test indicating the observed isolation-by-distance signal driven by among-group comparisons, but not within-group**. Trend line is for all comparisons. Solid and open dots are for the among- and within-group comparisons, respectively.

A significant isolation-by-distance (IBD) signal was observed when all Finnish populations were included in the analysis (R_XY _= 0.558; P < 0.001; figure 2). However, non-significant IBD patterns were observed within each of the three Finnish groups (R_XY _= -0.008, 0.233 and 0.314; all associated P > 0.05; within northern, Baltic and south-eastern groups, respectively). In addition, pooled within-group comparisons did not result in significant IBD signal (R_XY _= -0.153, P > 0.05). The Mantel test using ln distance provided the same conclusion as employing the raw geographic distance.

### Allele size permutation test

**Figure 3:**
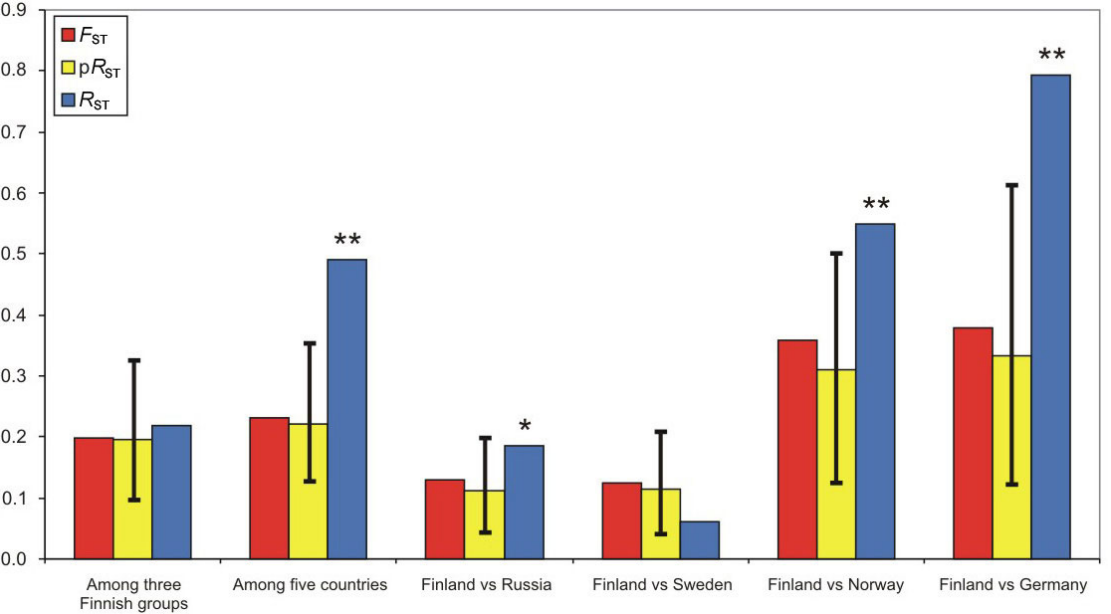
**Global *F*_ST_, p*R*_ST _and *R*_ST _estimated among three Finnish groups and among five countries**. The 95% confidence intervals are given for p*R*_ST_. One or two asterisks are indicated (for 0.01 ≤ P < 0.05 or P < 0.01, respectively) where global *R*_ST _was significant compared to the null distribution p*R*_ST_.

The allele size permutation test indicated that mutations at microsatellite loci did not significantly contribute to genetic differentiation among the three Finnish groups compared to genetic drift and migration. At the European scale, the permutation test indicated that mutations had significantly contributed to population differentiation (global multilocus *F*_ST _and *R*_ST _among 25 populations = 0.342 and 0.476, respectively; P < 0.001). In the among five countries comparison, the observed multilocus *R*_ST _also fell well above the upper limit of the 95% confidence interval (CI) of the null distribution of p*R*_ST _(global multilocus *F*_ST _and *R*_ST _among five countries = 0.230 and 0.492, respectively; P < 0.001, figure 3). Pairwise comparisons further indicated that *R*_ST _> p*R*_ST _when comparing Finland with Norway or Germany. The permutation test suggested that *R*_ST _was still significantly larger than p*R*_ST _when comparing Finland with Russia but the 95% CI of p*R*_ST _were not very different from the observed *R*_ST _(global multilocus *F*_ST _and *R*_ST _= 0.129 and 0.186, respectively; P = 0.044). However, *R*_ST _was not significantly larger than p*R*_ST _when comparing Finland with Sweden (global multilocus *F*_ST _and *R*_ST _= 0.124 and 0.062, respectively; P = 0.883). When the SweHol sample from the Baltic sea was excluded from the analyses because it essentially belongs to the same Baltic group as Kru, Pera, Kit and Iso *R*_ST _was still very similar to p*R*_ST _indicating the predominant role of genetic drift and migration over mutation when comparing Finnish and Swedish populations.

### Population prioritization and categorization for conservation

**Figure 4:**
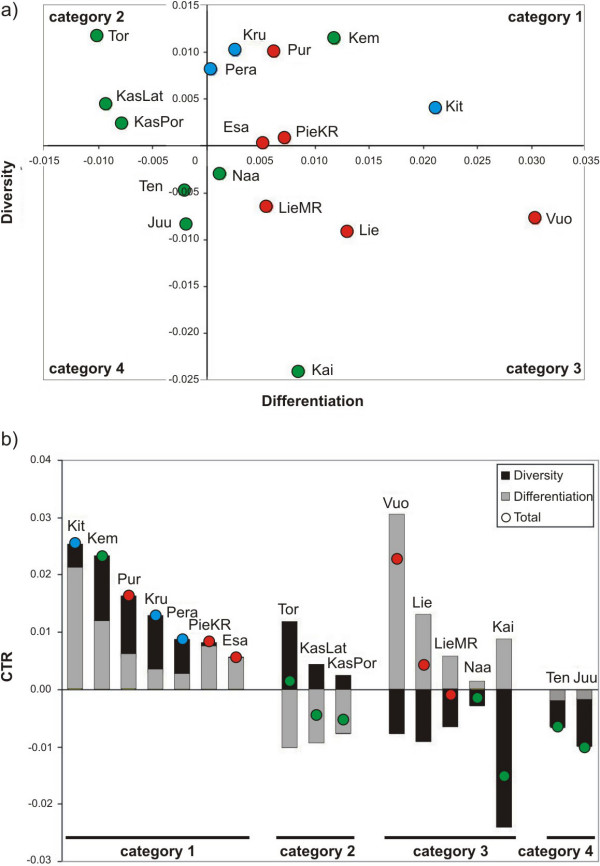
**Diversity and differentiation components of 17 Finnish grayling populations**. a) A 2D graph representing four different conservation categories of populations. Category 1 stands for high diversity-high differentiation, category 2 for high diversity-low differentiation, category 3 for low diversity-high differentiation and category 4 for low diversity-low differentiation. b) A bar graph indicating the categories and population prioritization (more important at left). Populations are colored differently according to the PCA and are coded as in table 1.

By plotting diversity and differentiation components calculated using the Contrib program, the following categories of populations were identified: 1) high diversity-high differentiation group, 2) high diversity-low differentiation group; 3) low diversity-high differentiation group and 4) low diversity-low differentiation group (figure 4a). Arguably, these categories largely reflect the relative roles of genetic drift and gene flow affecting the grayling populations and therefore can be useful for developing genetically justified conservation strategies for populations. The first category (high diversity-high differentiation group) contains seven populations that have high levels of diversity and exhibit differentiation higher than average. The second category (high diversity-low differentiation group) contains three populations that have a higher amount of diversity than average while the level of differentiation is rather low compared to the other populations. The third category (low diversity-high differentiation group) contains five populations that exhibit a lower amount of diversity than average and at the same time shows an increased differentiation component. The fourth category (low diversity-low differentiation group) contains two populations that exhibit somewhat lower genetic diversity as well as reduced differentiation compared to the average. For comparative purpose, the Contrib results are presented in a more traditional way by combining the diversity and differentiation components (figure 4b).

## Discussion

### Unanticipated population structuring

In accordance with other population genetic studies in European grayling [3,8,11,13,36,37] we found a generally high level of differentiation between the majority of populations. However, in contrast to our expectations and previous studies using microsatellites and mitochondrial DNA, we identified unanticipated signals of regional clustering of grayling populations in Finland. Such grouping was evident using both PCA and individual multi-locus genotype based analysis and further supported by analysis of molecular variance (AMOVA). The three population groups identified roughly correspond to separate geographic areas: the northern, Baltic and south-eastern regions. Nevertheless, all these three identified groups belong to a single European grayling mitochondrial lineage [9] and thus could be considered as one evolutionarily significant unit based on criteria *sensu *Moritz [38]. One of the reasons why the previous studies [9,13] of grayling in Finland did not detect such clustering is probably due to the low number of populations analyzed from the region. We observed high genetic differentiation between three population groups and moderate to high differentiation within the northern, Baltic and south-eastern groups. Significant IBD signal was identified when all Finnish populations were analyzed together while no significant relationship between genetic and geographic distances was found among populations within the three groups and pooled within-group comparisons. Thus, the overall IBD signal most likely derives from the structuring of Finnish populations into three groups rather than from concurrent gene flow between neighboring populations, even though the power to detect significant IBD signal was lower for within group comparisons. This is in accordance with other studies in European grayling demonstrating that inter-population dispersal is extremely limited even among neighboring populations [11,39,40].

Rather surprisingly, several different types of analyses strongly indicated that the Kitkajärvi population (Kit) groups together with the Baltic populations even though this population inhabits an inland lake that is relatively distant from the Baltic Sea (174 km) and, moreover, currently flows into the White Sea basin. However, it is known that Kitkajärvi (lake Kitka) had postglacially bifurcated outflow; the eastward channel drained into the Baltic sea via the system of Livojärvi (lake Livo) until around 8 400 years ago, and the westward channel flowed into the White Sea via Kitkajoki (river Kitka) as it still does [41]. The postglacial colonization of grayling into northern Europe has been estimated to have begun ca. 10 500 – 13 000 years ago [9] and thus we can hypothesize that the present Kitkajärvi grayling population was established through the historical waterway from the Baltic Sea more than 8 400 years ago. An alternative explanation is that there has been stocking activities of Baltic graying into Kitkajärvi, although there is no documented evidence supporting this scenario.

### Genetic diversity

Among salmonid fishes, European grayling is one of the least genetically variable species, exhibiting low diversity even across highly variable markers such as microsatellites. In Atlantic salmon (*Salmo salar*), brown trout (*Salmo trutta*), whitefish (*Coregonus hoyi*) and rainbow trout (*Oncorhynchus mykiss*), the mean expected heterozygosity measured at microsatellite loci commonly varies from 0.63 to 0.76 (e.g. [42-45]) while the mean expected heterozygosity in this study was as low as 0.41 and ranged from 0.20 to 0.63. Hence, either the effective population size, level of migration or microsatellite mutation rate in European grayling is smaller than in other salmonid populations. When looking at specific populations, NorLes from Norway had extremely low allelic richness (A_r _= 1.81) and low heterozygosity (H_e _= 0.24), which is in accordance with the demographic history of the population as it is known that NorLes was most likely established by a small number of individuals in 1880 [10]. However, we found that the natural population (Kai) in Inarijoki flowing to lake Inari showed even lower levels of diversity (A_r _= 1.56, H_e _= 0.20). As most of the study populations are from the area that was completely covered by an ice sheet during the last glacial period, it is possible that the low variability of grayling in northern Europe reflects postglacial colonization and subsequent founder effects [46,47]. However, the southernmost population from central Germany included in our study showed relatively low levels of variability (A_r _= 3.23, H_e _= 0.41) and other studies concentrating on the southern distribution range of European grayling have shown low diversity levels [2,7,8,37,48]. This strongly indicates that genetic drift and limited migration have a strong impact on diversity and population structure of grayling. However, as there is no estimator of the microsatellite mutation rate available for grayling one cannot exclude the possibility that the mutation rate in European grayling is lower than in other salmonid fishes, although it seems unlikely.

### The role of mutations contributing to population differentiation

Despite the clear separation of the three population groups in Finland there was no evidence that mutations have significantly contributed to the genetic differentiation among these groups. Thus, even though we observed relatively high differentiation between the three population groups, the allele permutation test indicated that patterns of differentiation are mostly driven by genetic drift and low migration rather than accumulation of new mutations. However, the allele size permutation test strongly suggested that mutations significantly contributed to population differentiation on a broader geographic scale.

### Population conservation categorization based on the role of different evolutionary forces

The "central dogma of conservation genetics" is that genetic variability is beneficial and therefore it is often assumed that increasing genetic variability enhances population survival [49]. Another important aspect of prioritizing populations for conservation is their genetic uniqueness measured at the molecular genetic or phenotypic level (e.g. evolutionary distinctiveness [50]). Hence for population conservation, it is relevant to evaluate both of these parameters but considerable controversy exists about the relative weights of diversity and uniqueness components (e.g [51]). Based on the strong clustering of Finnish populations, it is recommendable that national conservation efforts include populations from all three genetically distinct groups. In this study, we used an alternative approach that is still based on examining populations according to diversity and differentiation but instead of simply summing the diversity and uniqueness components based on rather subjective weighting, populations were categorized based on the predominant evolutionary forces acting on them. As a result, this approach is expected to be more objective compared to ranking solely on the summation of diversity and differentiation components based on arbitrary weights without losing crucial information about the relative roles of underlying evolutionary forces. For example, when simply combining diversity and differentiation components *sensu *Petit *et al*. [33], it is possible to get similar prioritization ranks for two populations with markedly different demographic histories – a population with low diversity-high differentiation can have a similar priority rank as another population with high diversity-low differentiation. It is clear that the first population could be severely affected by e.g. inbreeding depression while the second population might contain high genetic variability necessary for population survival and long-term evolution.

When examining diversity and differentiation components in grayling populations, we identified four categories where the relative importance of different evolutionary forces (namely drift and migration) varies. These categories are high diversity-high differentiation (category 1), high diversity-low differentiation (category 2), low diversity-high differentiation (category 3) and low diversity-low differentiation (category 4). From the conservation perspective, the populations falling into the high diversity-high differentiation category (e.g. Kit, Kem and Pur; from the Baltic, northern and south-eastern groups, respectively) may be less affected by genetic drift and migration as they represent large isolated populations. This high diversity-high differentiation category has the highest likelihood of containing unique genetic material. Notably, all three Baltic members fall into this category and given that grayling populations in the Baltic Sea are likely adapted to the brackish environment, they are hence highly relevant for conservation [52]. Populations that belong to the high diversity-low differentiation category (Tor, KasLat and KasPor) represent relatively large populations where the effect of drift is small, while migration may have some effect on the gene pool. We propose that populations in these two categories represent the top priority for conservation. Populations that belong to the low diversity-high differentiation category (e.g. Vuo, Lie and Kai) may represent small populations strongly affected by genetic drift. From a conservation genetic perspective, these populations have a lower probability of being able to evolve and survive in the future. Importantly, this does not necessarily mean that these populations cannot adapt to local conditions. For example, it has been shown that even in small grayling populations natural selection can have a predominant role over random genetic drift in affecting the fitness of phenotypic traits [10]. Populations that belong to the low diversity-low differentiation category (Ten and Juu) might be classified as a low conservation priority, as low differentiation reflects their lack of 'uniqueness' compared to other populations and low variability can additionally hinder adaptation in the future. However, it is also important to emphasize that these two populations have both the diversity and differentiation components quite close to the average across all populations. Taken together, the high levels of genetic differentiation observed among grayling populations here, as well as in other studies [3,4,8,11,37] clearly suggests that generally single populations should be the principal unit for conservation and management and thus population intermixing should be avoided. We also recommend using the proposed categorization strategy, taking into account the relative role of different evolutionary forces, as a basis for the conservation of grayling in Finland as well as of other species.

## Conclusion

This research provides genetic information on European grayling in its northern distribution range in order to assist country-scale conservation. We found unanticipated population structure of grayling, clustering into three groups largely corresponding to the northern, Baltic and south-eastern geographic areas of Finland and we recommend that these three groups should be used as the starting point for developing a national grayling conservation strategy. However, as the observed clusters extend beyond the borders of Finland, international co-operation for broader scale conservation management is warranted. We also found high levels of genetic differentiation among the groups and moderate to high differentiation within the groups. Such strong divergence indicates that single grayling populations should generally be recognized as separate management units. We also developed an alternative prioritization strategy in the conservation perspective by categorizing populations based on the evaluation of the relative role of various evolutionary forces affecting the indigenous gene pool. We envision that the proposed population categorization approach could be useful for a diverse range of organisms.

## Competing interests

The authors declare that they have no competing interests.

## Authors' contributions

AS carried out the molecular genetic work, statistical analyses and wrote the first draft of the manuscript. MTK performed genotyping in some populations. JP conducted the majority of the fieldwork. MTK, JP, AV and CRP co-designed the research theme. AV and CRP were involved in developing later drafts of the manuscript. All authors read and approved the final manuscript.

## Supplementary Material

Additional file 1 **Characteristics of microsatellite loci used in the study.** Primer concentration and summary statistics of microsatellite loci. Click here for file

Additional file 2 **Electropherograms of four individuals, each genotyped at 15 microsatellite loci in a single multiplex polymerase chain reaction.** Electropherograms generated by GeneMapper software. Click here for file

Additional file 3 **Pairwise genetic distance as measured with *R*_ST _(above the diagonal) and *F*_ST _(below the diagonal).** Pairwise genetic distance of the grayling populations as measured with *R*_ST _and *F*_ST_. Click here for file
