# Supplementary material for: Unanticipated population structure of European grayling in its northern distribution: implications for conservation prioritization
Source: Front Zool. 2009 Mar 30;6:6. doi: 10.1186/1742-9994-6-6 (PMC2676281; doi:10.1186/1742-9994-6-6)
Supplement: Additional file 1 — Characteristics of microsatellite loci used in the study. Primer concentration and summary statistics of microsatellite loci. [file 1742-9994-6-6-S1.doc]

### Additional file 1 –Characteristics of microsatellite loci used in the study.

| Locus information | |  | Amplification details | | |  | 25-population genetic diversity | | |
| --- | --- | --- | --- | --- | --- | --- | --- | --- | --- |
| Reference | Locus |  | Primer  Conc. (*μ*M) | Forward primer with 5' fluorescence dye | Reverse primer with 5’ tailing (bold letters) |  | Allele number | Allele range (bp) | H_e_ |
| Sušnik *et al*. (2000) | BFRO10 |  | 0.070 | FAM--5'--gga cgg agc cag cat cac | 5'--**gtt t**gc ccc cag gtt atc ata gct |  | 10 | 92-122 | 0.72 |
| Sušnik *et al*. (1999a) | BFRO5 |  | 0.050 | FAM--5'--cgc atc tgt atg aaa aac ct | 5'--**gtt t**tg gtt tgg tag gag ttt cgt |  | 12 | 112-142 | 0.41 |
| Snoj *et al*. (1999) | BFRO4^a^ |  | 0.034 | FAM--5'--gct cca gtg agg gtg acc ag | 5'--**gtt t**ag gcc act gat tga gca gag |  | 10 | 148-178 | 0.34 |
| GenBank acc. no. AF151370 | BFRO13 |  | 0.022 | FAM--5'--gat gta gtt gca ttg ctt gct ct | 5'--**gtt t**gg ctt tac cat tat cat atg agc |  | 16 | 195-247 | 0.82 |
| Sušnik *et al*. (1999a) | BFRO7 |  | 0.042 | FAM--5'--aga ccc cca aaa act atg ct | 5'--**gtt t**ct tca gca ggg gga gat aaa |  | 7 | 263-279 | 0.46 |
|  |  |  |  |  |  |  |  |  |  |
| GenBank acc. no. AF130411 | BFRO12 |  | 0.050 | NED--5'--tct gca cat cca aag cca tc | 5'--**gtt t**aa tct ctc tta atg aat cgt |  | 13 | 125-189 | 0.71 |
| Presa and Guyomard (1996) | Str85INRA |  | 0.403 | NED--5'--gga agg aag gga gaa agg t | 5'--gga aaa tca ata cta aca a |  | 8 | 165-197 | 0.24 |
| Bernatchez (1996) | Cocl23 |  | 0.183 | NED--5'--gca tta ggt cgt ttt gtg t | 5'--**gtt t**gc tgt atg agg ata gca ttc |  | 13 | 233-299 | 0.79 |
|  |  |  |  |  |  |  |  |  |  |
| Sušnik *et al*. (2000) | BFRO11 |  | 0.445 | PET--5'--cat ggt tga ttg tgg ggg ga | 5'--**gtt t**aa cat cct tac gcc cta gca |  | 14 | 84-116 | 0.74 |
| Estoup *et al*. (1993) | Str73INRA |  | 0.057 | PET--5'--cta ttc tgc ttg taa cta gac cta | 5'--**gtt t**cc tgg aga tcc tcc agc agg a |  | 10 | 133-165 | 0.61 |
| Scribner *et al*. (1996) | ONE2^a^ |  | 1.915 | PET--5'--cag gaa ttt aca gga ccc agg tt | 5'--ggt gcc aag gtt cag ttt atg tt |  | 55 | 205-397 | 0.88 |
|  |  |  |  |  |  |  |  |  |  |
| Sušnik *et al*. (1999b) | BFRO17 |  | 0.028 | VIC--5'--gcc cct ctg cta aac aca c | 5'--**gtt t**ct att ggg ttg agg tct gg |  | 6 | 111-125 | 0.38 |
| Sušnik *et al*. (1999b) | BFRO15 |  | 0.023 | VIC--5'--gac tca gtg aag aac taa agt aca | 5'--**gtt t**ga aaa gtt atg aag gtc aac cc |  | 13 | 142-190 | 0.58 |
| Sušnik *et al*. (1999b) | BFRO18 |  | 0.015 | VIC--5'--aga ggg gtc cag caa cat ca | 5'--**gtt t**gg gga acc agt cta aag cct |  | 18 | 171-205 | 0.84 |
| Olsen *et al*. (1998) | Ogo2 |  | 0.041 | VIC--5'--aca tcg cac acc ata agc at | 5'--**gtt t**cg act gtt tcc tct gtg ttg ag |  | 16 | 201-253 | 0.74 |

^a^ The amplified loci that were excluded before data analyses (see material and methods)

H_e_ Expected heterozygosity.

**References**

Bernatchez L: **Résau de suivi environnemental du complex la grande. Caractérisation génétique des formes naines et normales de grande corégone du réservoir Caniapiscau et du lac Sérigny à l’aide de marqueurs microsatellites.** Rapport présenté par l’université Laval à la vice-présidence Environnement et Collectivites d’Hydro-Québec; 1996.

Estoup A, Presa P, Krieg F, Vaiman D, Guyomard R: **(CT)_n_ and (GT)_n_ microsatellites - a new class of genetic markers for *Salmo trutta* L (Brown Trout).** *Heredity* 1993, **71:**488-496.

Olsen JB, Bentzen P, Seeb JS: **Characterization of seven microsatellite loci derived from pink salmon.** *Molecular Ecology* 1998, **7:**1087-1089.

Presa P, Guyomard R: **Conservation of microsatellites in three species of salmonids.** *Journal of Fish Biology* 1996, **49:**1326-1329.

Scribner KT, Gust JR, Fields RL: **Isolation and characterization of novel salmon microsatellite loci: Cross-species amplification and population genetic applications.** *Canadian Journal of Fisheries and Aquatic Sciences* 1996, **53:**833-841.

Snoj A, Susnik S, Pohar J, Dovc P: **The first microsatellite marker (BFRO 004) for grayling, informative for its Adriatic population.** *Animal Genetics* 1999, **30:**74-75.

Sušsnik S, Snoj A, Dovc P: **Microsatellites in grayling (*Thymallus thymallus*): comparison of two geographically remote populations from the Danubian and Adriatic river basin in Slovenia.** *Molecular Ecology* 1999a, **8:**1756-1758.

Sušnik S, Snoj A, Dovc P: **A new set of microsatellite markers for grayling: BFRO014, BFRO015, BFRO016, BFRO017 and BPRO018.** *Animal Genetics* 1999b, **30:**478-478.

Susnik S, Snoj A, Jesensek D, Dovc P: **Rapid communication: Microsatellite DNA markers (BFRO010 and BFRO011) for grayling.** *Journal of Animal Science* 2000, **78:**488-489
