# Supplementary figures and images for: Unanticipated population structure of European grayling in its northern distribution: implications for conservation prioritization
Source: Front Zool. 2009 Mar 30;6:6. doi: 10.1186/1742-9994-6-6 (PMC2676281; doi:10.1186/1742-9994-6-6)

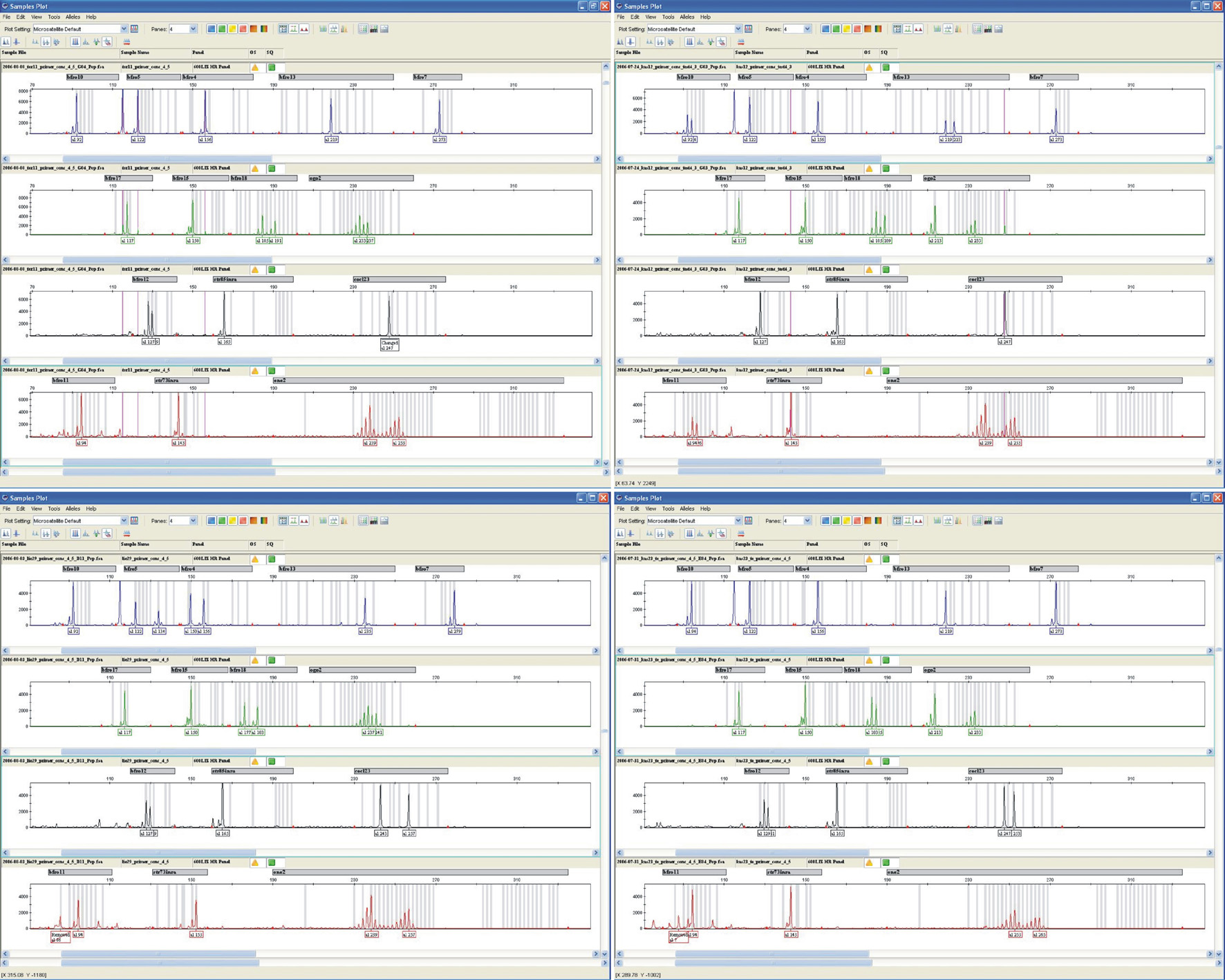

Supplement: Additional file 2 — Electropherograms of four individuals, each genotyped at 15 microsatellite loci in a single multiplex polymerase chain reaction. Electropherograms generated by GeneMapper software. [file 1742-9994-6-6-S2.jpeg]
