# Supplementary material for: Unanticipated population structure of European grayling in its northern distribution: implications for conservation prioritization
Source: Front Zool. 2009 Mar 30;6:6. doi: 10.1186/1742-9994-6-6 (PMC2676281; doi:10.1186/1742-9994-6-6)
Supplement: Additional file 3 — Pairwise genetic distance as measured with RST (above the diagonal) and FST (below the diagonal). Pairwise genetic distance of the grayling populations as measured with RST and FST. [file 1742-9994-6-6-S3.doc]

### Additional file 3 - Pairwise genetic distance as measured with *R*_ST_ (above the diagonal) and *F*_ST_ (below the diagonal). Populations are coded as in table 1.

|  | Northern Finland | | | | | | | |  | The Baltic | | | |  | South-eastern Finland | | | | | | |  | Other countries | | | | | |
| --- | --- | --- | --- | --- | --- | --- | --- | --- | --- | --- | --- | --- | --- | --- | --- | --- | --- | --- | --- | --- | --- | --- | --- | --- | --- | --- | --- | --- |
|  | Ten | Naa | Kai | KasPor | KasLat | Tor | Juu | Kem |  | Kit | Pera | Kru | Iso |  | Lie | LieMR | PieKR | Rau | Pur | Esa | Vuo |  | RusJus | SweVin | SweHol | SweVat | NorLes | GerEge |
| Ten | 0 | 0.087 | 0.248 | 0.088 | 0.119 | 0.027 | 0.124** | 0.117 |  | 0.245 | 0.311 | 0.285 | 0.461 |  | 0.329 | 0.382 | 0.493 | 0.255 | 0.206 | 0.422 | 0.693 |  | 0.279 | 0.271 | 0.243 | 0.510 | 0.797* | 0.798** |
| Naa | 0.120 | 0 | 0.195 | 0.225 | 0.207* | 0.112 | 0.101 | 0.140 |  | 0.292 | 0.354 | 0.316 | 0.487 |  | 0.372 | 0.410 | 0.487* | 0.229 | 0.185 | 0.413 | 0.661* |  | 0.247 | 0.232 | 0.279 | 0.513 | 0.784** | 0.792** |
| Kai | 0.229 | 0.208 | 0 | 0.335 | 0.277* | 0.176 | 0.180 | 0.185 |  | 0.343 | 0.478 | 0.414 | 0.572 |  | 0.412 | 0.437 | 0.561 | 0.293 | 0.202 | 0.423 | 0.722 |  | 0.272 | 0.248 | 0.320 | 0.563 | 0.831 | 0.800 |
| KasPor | 0.110 | 0.196 | 0.354 | 0 | 0.018 | 0.036 | 0.238* | 0.147 |  | 0.171 | 0.219 | 0.238 | 0.389 |  | 0.248 | 0.322 | 0.385 | 0.209 | 0.137 | 0.298 | 0.590 |  | 0.297 | 0.278 | 0.121 | 0.472 | 0.757** | 0.747* |
| KasLat | 0.089 | 0.174 | 0.293 | 0.009 | 0 | 0.058 | 0.232** | 0.131 |  | 0.174 | 0.204 | 0.226 | 0.377 |  | 0.227 | 0.295 | 0.299 | 0.144 | 0.105 | 0.244 | 0.506 |  | 0.274 | 0.264 | 0.089 | 0.463 | 0.727* | 0.752* |
| Tor | 0.067 | 0.164 | 0.254 | 0.035 | 0.028 | 0 | 0.074 | 0.105 |  | 0.258 | 0.277 | 0.284 | 0.454 |  | 0.297 | 0.356 | 0.444 | 0.232 | 0.166 | 0.366 | 0.613 |  | 0.228 | 0.275 | 0.213 | 0.493 | 0.751** | 0.805** |
| Juu | 0.076 | 0.159 | 0.198 | 0.133 | 0.083 | 0.069 | 0 | 0.140 |  | 0.341 | 0.429 | 0.382 | 0.549 |  | 0.397 | 0.438 | 0.540* | 0.292 | 0.201 | 0.434 | 0.702 |  | 0.251 | 0.288 | 0.317 | 0.532 | 0.807* | 0.802** |
| Kem | 0.148 | 0.203 | 0.281 | 0.103 | 0.095 | 0.092 | 0.161 | 0 |  | 0.289 | 0.320 | 0.322 | 0.452* |  | 0.333 | 0.392 | 0.452* | 0.188 | 0.136 | 0.391 | 0.609* |  | 0.117 | 0.210 | 0.203 | 0.491 | 0.732** | 0.741** |
| Kit | 0.281 | 0.251 | 0.364 | 0.277 | 0.270 | 0.262 | 0.321 | 0.231 |  | 0 | 0.078 | 0.112 | 0.061* |  | 0.138 | 0.208 | 0.284 | 0.220 | 0.213 | 0.267 | 0.445 |  | 0.349 | 0.293 | 0.095 | 0.289 | 0.599* | 0.737* |
| Pera | 0.272 | 0.231 | 0.406 | 0.226 | 0.212 | 0.204 | 0.273 | 0.161 |  | 0.160 | 0 | -0.007 | 0.181 |  | 0.074 | 0.094 | 0.343 | 0.203 | 0.202 | 0.285 | 0.527 |  | 0.459 | 0.289 | 0.188 | 0.241 | 0.683** | 0.740* |
| Kru | 0.267 | 0.235 | 0.381 | 0.256 | 0.238 | 0.225 | 0.276 | 0.181 |  | 0.174 | 0.002 | 0 | 0.193 |  | 0.105 | 0.107 | 0.343 | 0.201 | 0.234 | 0.316 | 0.522 |  | 0.441 | 0.289 | 0.232 | 0.233 | 0.653* | 0.767** |
| Iso | 0.376 | 0.325 | 0.473 | 0.349 | 0.352 | 0.340 | 0.415 | 0.303 |  | 0.040 | 0.239 | 0.245 | 0 |  | 0.208 | 0.270 | 0.422 | 0.337 | 0.358 | 0.425 | 0.590 |  | 0.540 | 0.443 | 0.261 | 0.333 | 0.680 | 0.750* |
| Lie | 0.419 | 0.440 | 0.510 | 0.373 | 0.357 | 0.335 | 0.423 | 0.258 |  | 0.354 | 0.322 | 0.329 | 0.420 |  | 0 | 0.029 | 0.358 | 0.222 | 0.232 | 0.294 | 0.493 |  | 0.442 | 0.300 | 0.228 | 0.291 | 0.648 | 0.768* |
| LieMR | 0.408 | 0.428 | 0.487 | 0.381 | 0.357 | 0.323 | 0.407 | 0.244 |  | 0.364 | 0.329 | 0.328 | 0.438 |  | 0.032 | 0 | 0.395 | 0.250 | 0.296 | 0.340 | 0.517 |  | 0.505 | 0.342 | 0.318 | 0.305 | 0.654 | 0.790** |
| PieKR | 0.395 | 0.362 | 0.468 | 0.293 | 0.295 | 0.310 | 0.387 | 0.273 |  | 0.274 | 0.206 | 0.243 | 0.317 |  | 0.315 | 0.348 | 0 | 0.163 | 0.225 | 0.154 | 0.200 |  | 0.539** | 0.478 | 0.205 | 0.479 | 0.683** | 0.771** |
| Rau | 0.308 | 0.268 | 0.383 | 0.247 | 0.243 | 0.246 | 0.311 | 0.216 |  | 0.256 | 0.191 | 0.207 | 0.304 |  | 0.320 | 0.332 | 0.173 | 0 | 0.073 | 0.180 | 0.325 |  | 0.277 | 0.210 | 0.139 | 0.356 | 0.597** | 0.704** |
| Pur | 0.353 | 0.327 | 0.437 | 0.258 | 0.257 | 0.265 | 0.348 | 0.202 |  | 0.214 | 0.160 | 0.190 | 0.275 |  | 0.237 | 0.268 | 0.181 | 0.170 | 0 | 0.113 | 0.304 |  | 0.154 | 0.193 | 0.074 | 0.364 | 0.578* | 0.715** |
| Esa | 0.394 | 0.381 | 0.480 | 0.324 | 0.320 | 0.322 | 0.405 | 0.266 |  | 0.253 | 0.208 | 0.220 | 0.320 |  | 0.287 | 0.313 | 0.184 | 0.191 | 0.069 | 0 | 0.143 |  | 0.409 | 0.361 | 0.154 | 0.422 | 0.592* | 0.763** |
| Vuo | 0.549 | 0.503 | 0.632 | 0.485 | 0.478 | 0.466 | 0.545 | 0.423 |  | 0.431 | 0.388 | 0.396 | 0.480 |  | 0.418 | 0.455 | 0.263 | 0.286 | 0.282 | 0.292 | 0 |  | 0.674** | 0.596 | 0.369 | 0.576 | 0.725** | 0.800** |
| RusJus | 0.289 | 0.231 | 0.356 | 0.285 | 0.272 | 0.234 | 0.298 | 0.212 |  | 0.233 | 0.230 | 0.231 | 0.279 |  | 0.368 | 0.365 | 0.336 | 0.233 | 0.228 | 0.304 | 0.457 |  | 0 | 0.176 | 0.255 | 0.542 | 0.749* | 0.736** |
| SweVin | 0.346 | 0.267 | 0.423 | 0.342 | 0.350 | 0.336 | 0.382 | 0.272 |  | 0.289 | 0.258 | 0.269 | 0.340 |  | 0.393 | 0.383 | 0.385 | 0.284 | 0.300 | 0.340 | 0.466 |  | 0.257 | 0 | 0.272 | 0.416 | 0.665* | 0.724* |
| SweHol | 0.221 | 0.211 | 0.328 | 0.162 | 0.165 | 0.165 | 0.251 | 0.101 |  | 0.114 | 0.090 | 0.107 | 0.174 |  | 0.253 | 0.267 | 0.179 | 0.151 | 0.094 | 0.145 | 0.322 |  | 0.173 | 0.204 | 0 | 0.397 | 0.629** | 0.699** |
| SweVat | 0.483 | 0.480 | 0.578 | 0.430 | 0.436 | 0.440 | 0.513 | 0.341 |  | 0.393 | 0.383 | 0.379 | 0.417 |  | 0.473 | 0.489 | 0.423 | 0.373 | 0.382 | 0.417 | 0.524 |  | 0.441 | 0.395 | 0.280 | 0 | 0.585 | 0.755** |
| NorLes | 0.593 | 0.582 | 0.695 | 0.586 | 0.572 | 0.533 | 0.609 | 0.492 |  | 0.492 | 0.499 | 0.471 | 0.551 |  | 0.580 | 0.588 | 0.500 | 0.444 | 0.457 | 0.474 | 0.574 |  | 0.552 | 0.443 | 0.404 | 0.450 | 0 | 0.759* |
| GerEge | 0.562 | 0.553 | 0.639 | 0.517 | 0.518 | 0.514 | 0.579 | 0.447 |  | 0.473 | 0.467 | 0.459 | 0.505 |  | 0.510 | 0.539 | 0.487 | 0.447 | 0.425 | 0.472 | 0.550 |  | 0.495 | 0.480 | 0.371 | 0.424 | 0.576 | 0 |

^*^ (significant; 0.01≤P<0.05) and ^**^ (highly significant; P<0.01) indicated under one-sided test of H_1_: *R*_ST_ > permuted *R*_ST_ (theoretically referred as *F*_ST_)
